# Deadline-Aware Energy-Efficient Query Scheduling in Wireless Sensor Networks with Mobile Sink

**DOI:** 10.1155/2013/834653

**Published:** 2013-05-29

**Authors:** Murat Karakaya

**Affiliations:** Department of Computer Engineering, Atilim University, Incek, 06836 Ankara, Turkey

## Abstract

Mobile sinks are proposed to save sensor energy spent for multihop communication in transferring data to a base station (sink) in Wireless Sensor Networks. Due to relative low speed of mobile sinks, these approaches are mostly suitable for delay-tolerant applications. In this paper, we study the design of a query scheduling algorithm for query-based data gathering applications using mobile sinks. However, these kinds of applications are sensitive to delays due to specified query deadlines. Thus, the proposed scheduling algorithm aims to minimize the number of missed deadlines while keeping the level of energy consumption at the minimum.

## 1. Introduction

A Wireless Sensor Network (WSN) can be defined as a network of sensor nodes deployed to monitor a field with wireless communication capability and base stations (sinks) to gather information from sensors for uploading to a remote central. Usually sensor nodes are powered by unrechargeable batteries. When a sensor depletes its battery, it becomes nonfunctional which can affect the connectivity and correctness of WSN. Therefore, energy consumption is a crucial factor affecting the life time of a WSN.

Various energy conservation approaches have been proposed and implemented so far to maximize network lifetime as surveyed in [1]. One common way to decrease the energy consumption in the communications between sensors and static sink (SS) is using multihop forwarding instead of direct connection. Multihop communication has the advantage of using low power in transmitting data to a nearby sensor. However, one of the main problems of applying Multihop paradigm in WSN is that the sensors around SS deplete their energy very fast due to high forwarding data traffic. When that occurs, the sink becomes unreachable and WSN is nonfunctional. As an alternative to Multihop communication, researchers have proposed to mobilize sinks to collect data from sensors [2]. These mobile sinks (MSs) are capable of moving in the monitored field and contacting sensors using either one hop (direct) or limited number of hops communication methods. As a result, the required energy for transferring data from sensors to sink is reduced considerably and the life time of WSN is extended significantly [3]. Unfortunately, this method suffers from relatively high delay time in uploading data from sensors due to lower speed of MS compared to speed of radio communication. Therefore, this approach is suitable for delay tolerant applications and not a good option for real or near real time applications such as location-based querying or target tracking [4].

In this paper, we present a novel way of using MS for a near real time application, namely, query-based data gathering with deadlines, by trading off delay in response with energy consumption in Multihop communication. In this class of applications, location-based queries are submitted to WSN, and responses should be collected before the specified deadline expires. For this reason, we design a query scheduling algorithm to exploit MS deterministic mobility for saving energy in communication and to exploit speed of Multihop communication for minimizing delay caused by slow MS motion, whenever any of them is feasible. Thus, our algorithm balances the system throughput and energy consumption by optimizing the number of hops and duration of response time. The algorithm is very simple yet successful and applicable to the situations where either controlling MS moves is not possible (e.g., geographic conditions) or feasible (e.g., attached to a public transport vehicle).

The paper is organized as follows: related work is presented in Section 2.  Section 3 provides the details of WSN model and the proposed query scheduling algorithm. Simulation model and results of simulation tests are discussed in Section 4. Finally, in Section 5 conclusion and future work direction are presented.

## 2. Related Work

The possible ways of initiating data transfer from sensors to a sink can be categorized into four classes: event-driven, time-driven, query-based, and hybrid [5]. In event-driven data collection approach, data needs to be collected whenever a specific event is detected and then forwarded to a sink (base station). Similarly, query-based data collection is triggered upon receiving a user's query, and response should be routed to a sink. On the other hand, in a time-driven approach, data collected by sensors is transferred to a sink periodically. As a last option, one can combine the above approaches to create hybrid approaches.

For any data collection approach, sensor readings should be transferred to some sink which can be classified as static sink (SS) and mobile sink (MS) according to mobility. Sink mobility affects the communication pattern between sensor and sink [6]. When static sink is used, sensors mostly depend on Multihop communication to forward their data to the sink. However, if sink can move, it can visit sensors and collect data from them directly.

MS mobility patterns can be listed as controlled, random, or deterministic [6]. In controlled mobility, MS next stop can be decided dynamically by an algorithm depending on some network or performance parameters. However, MS can follow some paths constructed by selecting next sensor randomly with a distribution probability in a random mobility model. In some other cases, MS moves deterministically on predefined or predictable paths (roads, railways, etc.) in regular time intervals.

In this work, we focus on query-based data collection in WSN using mobile sink with deterministic mobility pattern. Likewise, in [7, 8] the authors propose a query propagation and collection method minimizing energy and time using MS. They show that selecting the shortest path for delivering response packet is very important to minimize the total energy spending for the query. They calculate an optimum location on the path to submit a query such that query packet would arrive to target sensor, and then response would follow the shortest path to reach to the collect point just before MS arrives. Unfortunately, in the work they do not consider query deadlines, and their solution cannot be easily applied to handle the deadlines. Moreover, they assume that the target sensor location should always be ahead of MS moving direction. Therefore, if a target sensor is located on the other direction of the MS move, the proposed method cannot calculate any collection point at all. In reality, target sensor can be located at any place in the monitored area. In our solution, we propose a query scheduling algorithm to minimize the energy consumption and meet query deadlines for more realistic and generic scenario.

In another similar work, authors model a WSN in which sensors store collected data in their finite memory [9]. MS collects data from these sensors via one-hop communication pattern and frees their memory. If MS is late and sensor memory gets full, the sensor removes all the readings from memory and restarts to collect data. The task is to schedule MS to visit each sensor before the sensor memory overflows with the collected data. Thus, there is a deadline for visiting each sensor. In their solution, assuming that the initial capacity of each sensor memory and sensor sampling rates are known, the authors attempt to create an MS route such that MS would be able to visit each sensor before an overflow occurs. In our case, we aim to create energy-optimized paths for packets, while the queries to sensors are random. For MS mobility model, they assume that MS has a controlled mobility, while we assume it to be a deterministic mobility.

There are other query based data collection approaches which mainly focus on designing a high level interface for query—response interactions between application and sensors. Unfortunately, these approaches do not work on details of underlying network topology, communication requirements, and energy consumption issues [5].

## 3. Deadline-Aware Energy-Efficient Query Scheduling Algorithm

The *Deadline-Aware Energy-Efficient Query Scheduling Algorithm* (DES) has twofold objective: providing responses before the given deadlines and consuming minimum possible amount of sensor energy in the Multihop communication. Below we first present the WSN model and the problem and then provide the proposed solution details.

### 3.1. WSN Model and Problem Definition

**Figure 1:**
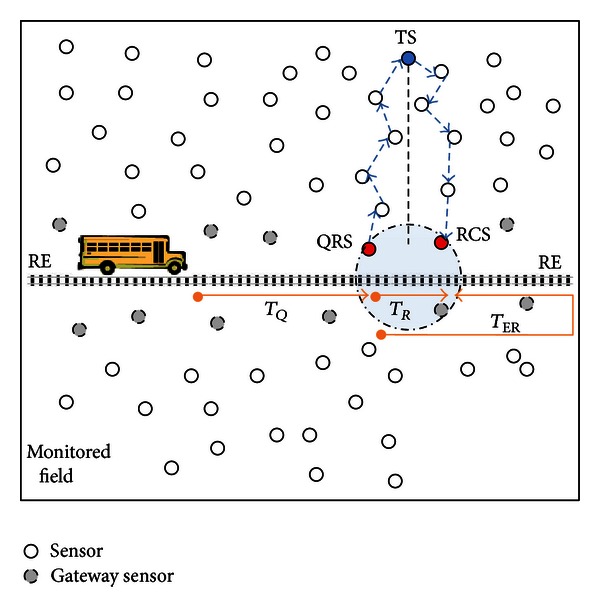
Assumed WSN model.

We assume that a WSN has been deployed for monitoring some environmental changes such as heat and mobility, as shown in Figure 1. WSN has three important components: sensor nodes, mobile sink, and Multihop routing protocol. In this model, each sensor node has its own limited memory to store the readings, and the location of the sensors is known. Underlying Multihop routing protocol can route messages from a source node to a destination node via a shortest path. Mobile sink consistently moves back and forth with a fixed velocity on a predefined route between two *Route Ends* (RE). Actually, MS could be embedded onto a regularly moving object such as public transportation vehicle (bus, train, or ship).

Any sensor node which is onehop away from the route can directly communicate with passing by MS. These sensors are called *Gateway Sensors* (GSs). Similarly, MS can forward a query to a target sensor by submitting it to any GS around itself. Via direct connection WSN central management authority can send queries to MS and demand responses to be uploaded. In these queries the *Target Sensor* (TS) and the deadline of the response are specified. Query deadline is the time before which the query should be executed and the response should be delivered to the remote central. The queries with different deadlines arrive dynamically as MS keeps moving on the path.

Whenever the remote central initiates a query, MS attempts to create a schedule such that the communication cost (energy consumption) for forwarding messages would be minimum and response would be uploaded before the given deadline (the algorithm can be run by remote central or MS. In this work we assume that MS runs the algorithm whenever it receives a query). A schedule is composed of *Query Release Sensor* (QRS) to inject a query into the WSN and *Response Collect Sensor* (RCS) to collect the response.

**Figure 2:**
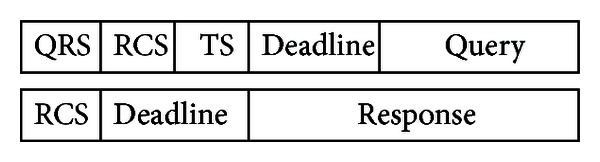
Query and response message content.

While preparing schedule, MS keeps moving and releases queries to related QRS when it is in one-hop proximity of QRS. This trip, from current MS location when it received the query to QRS location, is named *Query Release Trip* (*T*_*Q*_). WSN communication protocol takes care of forwarding the query message from QRS to *Target Sensor* (TS). TS processes the query, prepares the response message, and forwards it to RCS (query and response message contents are given in Figure 2). Intermediate sensors in the routing path drop any messages whose deadline are already expired.

As MS is passing by RCS, it attempts to collect the response message from the sensor. The trip from QRS to RCS is called *Response Collect Trip* (*T*_*R*_). If the response message has already arrived to RCS, MS can collect and upload it to remote central. On the other hand, if the response message has not arrived at RCS yet, MS takes *an Extended Response Collect Trip* (*T*_ER_) by moving to the route end (RE) and comes back to RCS once more. Thus, MS tries to collect the response message from RCS for the second time.

To meet the deadline in the WSN model given above, we need to ensure that MS reaches RCS before deadline and the response message reaches RCS before MS. Similarly to minimize the energy consumption in transferring query and response packages, we have to select QRS and RCS from GS such that they have the shortest path (minimum number of hops away) from the target sensor. However, these two conditions could not be always satisfied. Thus, the main scheduling problem is to find QRS and RCS such that MS can obtain the response before the deadline and routing costs the minimum energy consumption. Below we provide the details of Deadline-Aware Energy-Efficient Query Scheduling (DES).

### 3.2. Solution

 DES first attempts to construct *Least Energy Consuming Schedule* (LECS) using the shortest paths between QRS-TS and TS-RCS. If it is not possible due to deadline or message routing, it can attempt to create either *Optimum Energy Consuming Schedule* (OECS) keeping TS-RCS path shortest but extending QRS-TS path or *Maximum Energy Consuming Schedule* (MECS) modifying both TS-RCS and QRS-TS paths to gain more time. When none of the routing paths are feasible, DES drops the request immediately.

As summarized in Algorithms 1, 2, and 3, DES considers MS current location (*L*_MS_), movement direction (Dir_MS_), and speed (Speed_MS_) along with route ends location (*L*_RE_), target sensor location (*L*_TS_), query size (Size_*Q*_), response size (Size_*R*_), data transfer rate (Rate_Data_), query processing time (Proc_*Q*_), expected numbers of hops from QRS to destination (Hop_*D*_), expected number of hops from destination to RCS (Hop_RCS_), and query deadline (Deadline_*Q*_) for creating feasible schedules.

The details of creating schedules are given below.

#### 3.2.1. Constructing LECS

**Figure 3:**
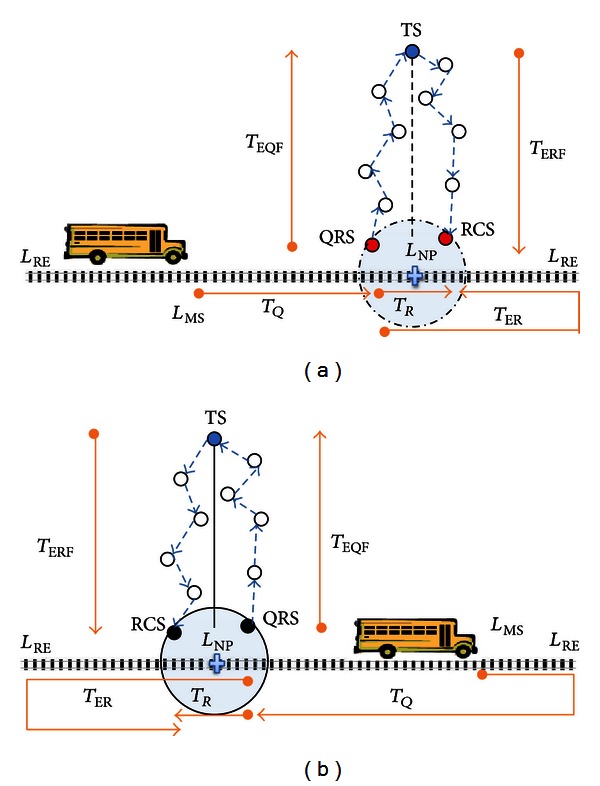
Least Energy Consuming Schedule when MS moves in the direction of TS (a) and when it moves in the other direction (b).

If deadline permits to create the shortest routing path between QRS to RCS via TS, the energy saving would be maximized since the number of hops are expected to be minimum as seen in Figure 3. Thus, LECS attempts to choose QRS and RCS among the gateway sensors which are nearest to TS. For this reason in Algorithm 1, the nearest point location (*L*_NP_) on the MS route to *L*_TS_ is calculated, and one-hop away gateway sensors around it are candidates for being *QRS* or *RCS*. If there is only one candidate sensor it is selected as both QRS and RCS. However, if there are more than one, DES selects the sensors which MS will contact first as QRS and other sensor which MS will contact latest as RCS. The reason for this selection is to maximize the chances of receiving the response message before MS arrives at RCS.

After deciding QRS and RCS, LECS checks several parameters to see if the query deadline can be met with the current schedule. The duration of Query Release Trip Time (*T*_*Q*_), Response Collect Trip Time (*T*_*R*_), and Extended Response Collect Trip Time (*T*_ER_) is computed as in (1), (2), and (3), respectively (MS movement direction is important factor for calculating *T*_*Q*_ (see Figure 3)). Moreover, *Expected Query Forwarding Time* (*T*_EQF_), time required to forward a query message from *L*_QRS_ to *TS*, and *Expected Query Processing and Response Forwarding Time* (*T*_ERF_), time needed to process the query and forward the response message to *RCS*, are estimated as in (4) and (5), respectively. In these equations, we estimated the number of hops for given two locations using the sensor density, communication range, and the bandwidth.

After calculating all these parameters, LECS first checks if MS has enough time to reach RCS location before the deadline. If deadline allows, MS should ensure the time needed for query forwarding and processing, and response forwarding would be less than trip time to RCS. In some cases response message would be late to arrive at RCS, and as a second chance, we might allow MS to move up to RE and come back to RCS. When response can reach RCS before the extended response collect trip time, we should check if deadline does not still expire (one can suggest that even in an extended trip time it is not enough, and deadline does not expire; therefore, MS can execute another tour on the path back to RCS once more. Since the communication speed is much more than the MS speed, we ignore this case).

According to all these conditions we either schedule QRS and RCS successfully, or we call other scheduling algorithms to calculate alternative paths. If LECS algorithm fails to create a shortest routing path due to late arrival of response message to RCS, it calls OECS to select an alternative QRS (AQRS) such that MS can release query earlier and WSN would have more time to route the response message to RCS. On the other hand when LECS algorithm fails because of deadline expiration before MS finishes collect trip, MECS algorithm is called for choosing alternative QRS (AQRS) and alternative RCS (ARCS) such that MS and the response message would meet at ARCS before the deadline. Consider

(1) $$\begin{array}{c} T_{Q}=\{\begin{array}{cc} \frac{\text{Dis}\left(L_{\text{MS}},L_{\text{QRS}}\right)}{\text{Spee}{\text{d}}_{\text{MS}}} & \\ \;\text{if}\;\text{Di}{\text{r}}_{\text{MS}}\;\text{is}\;\text{towards}\;\text{QRS} & \\ \frac{\text{Dis}\left(L_{\text{MS}},L_{\text{RE}}\right)+\text{Dis}\left(L_{\text{RE}},L_{\text{QRS}}\right)}{\text{Spee}{\text{d}}_{\text{MS}}} & \\ \;\text{if}\;\text{Di}{\text{r}}_{\text{MS}}\;\text{is}\;\text{reverse}\;\text{direction}\;\text{of}\;\text{QRS}, & \end{array} \end{array}$$

(2) $$\begin{array}{c} T_{R}=\frac{\text{Dis}\left(L_{\text{QRS}},L_{\text{RCS}}\right)}{\text{Spee}{\text{d}}_{\text{MS}}}, \end{array}$$

(3) $$\begin{array}{c} T_{\text{ER}}=\frac{\text{Dis}\left(L_{\text{QRS}},L_{\text{RE}}\right)+\text{Dis}\left(L_{\text{RE}},L_{\text{RCS}}\right)}{\text{Spee}{\text{d}}_{\text{MS}}}, \end{array}$$

(4) $$\begin{array}{c} T_{\text{EQF}}=\frac{{\text{Size}}_{Q}}{\text{Rat}{\text{e}}_{\text{Data}}}*{\text{Hop}}_{D}, \end{array}$$

(5) $$\begin{array}{c} T_{\text{ERF}}=\frac{{\text{Size}}_{R}}{\text{Rat}{\text{e}}_{\text{Data}}}*{\text{Hop}}_{\text{RCS}}. \end{array}$$

#### 3.2.2. Constructing OECS

**Figure 4:**
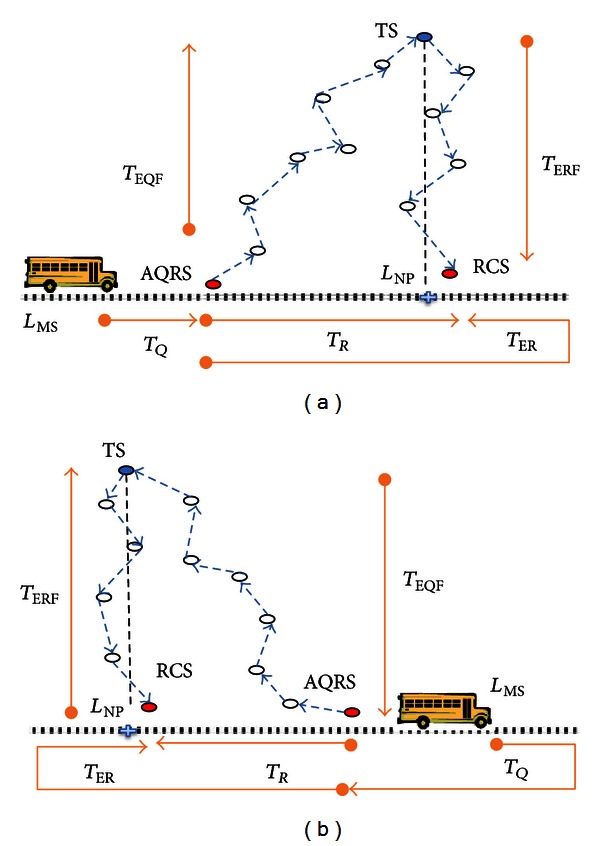
Optimum energy consuming schedule when MS moves in the direction of TS (a) and when it moves in the other direction (b).

As seen in Algorithm 2, OECS algorithm searches an alternative routing path when response message is late to arrive at RCS before MS passes by even though deadline allows MS to reach RCS. Therefore, OECS algorithm relocates QRS such that query message forwarding would begin earlier than before and reply message can arrive to RCS on time with optimum energy consumption in Multihop communications (see Figure 4). Since the response data size is expected to be larger than that of query, to save more energy in routing messages, response forwarding path should be kept shortest in the first place. Given densely and uniformly deployed sensors, we may construct minimum energy consuming path for forwarding response messages by creating a shortest path between TS and RCS, as in LECS algorithm. As a result, optimum route can be constructed if we can construct a routing path such that query forwarding would take some more hops, but response forwarding takes still the least number of hops, and response would be ready for MS to collect [7, 8].

Using RCS selected by LECS algorithm, OECS algorithm calculates alternative QRS (AQRS) location between *L*_MS_ and *L*_NP_ such that total time required for *T*_EQF_ and *T*_ERF_ would be less or equal to response collect trip time (*T*_*R*_). If such a location is feasible, AQRS is selected among gateway sensors one hop away from this location. Otherwise, we can select the nearest sensor from *L*_MS_ as AQRS and test if an extended response collect trip can be run before deadline, and result would be ready at RCS before MS reaches it. If this option fails as well, we call MECS to create an alternative schedule.

#### 3.2.3. Constructing MECS

As discussed above, LECS attempts to use minimum energy by constructing shortest routing paths for query and response message delivery, whereas OECS consumes least energy for response messages but more energy for delivering query to gain time. Whenever these two algorithms fail to create a feasible solution, as a last resort, they call MECS algorithm. The routing path constructed by MECS algorithm costs more energy to gain time by attempting to select an alternative QRS (AQRS) as well as an alternative RCS (ARCS) in the hope that response message can be reachable by MS before the deadline.

**Figure 5:**
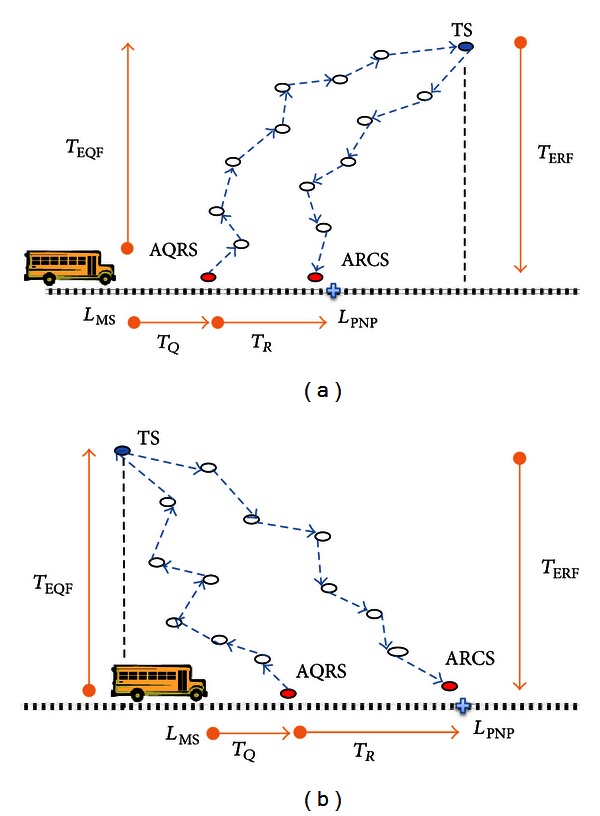
Most Energy Consuming Schedule when MS moves in the direction of TS (a) and when it moves in the other direction (b).

As shown in Figure 5, MECS first decides the possible nearest point (*L*_PNP_) on the route to TS where MS can get before deadline finishes. The sensors in the vicinity of *L*_PNP_ are candidates for alternative RCS (ARCS). Then alternative QRS (AQRS) location is calculated such that response message arrives to ARCS before MS.

Considering deadline if such AQRS location is not available, MECS algorithm recalculates AQRS and ARCS locations such that the distance between these two locations would be the largest. We hope that while MS moves from AQRS to ARCS, the query and reply message can be forwarded up to ARCS before deadline. If MECS algorithm again fails to find such AQRS and ARCS locations, the query will be rejected.

## 4. Simulation Model and Results

This section presents the evaluation of Energy-Efficient Deadline-Aware Scheduling with respect to several performance metrics and two other scheduling methods.

### 4.1. Simulation Model

Table 1 summarizes important simulation parameters and their default values which generally apply to those used in similar studies such as [7, 8, 10]. Below we discuss each simulation parameter in detail.

*WSN*. We assume a realistic deployment area of 1000 × 500 meters. We consider random topology in which sensors are deployed randomly on the monitored field. MS moves on a fixed route located in the middle of monitored field from east to west.

*Sensors*. The number of sensors is 1500 as default. The radio range of sensors is set to be 50 meters with a data rate of 256 Kb/s. We measure the energy consumed at the sensor network as an absolute value in Joules. Each sensor begins with a full battery of 0.1 joule and dissipates 50 nJ per bit of battery energy for transferring data packages. When the sensor is idle the energy consumption is assumed to be 40 nJ/s. Query processing takes 0.1 seconds and costs 100 nJ.

*Simulation*. Each simulation runs 15 simulated hours, and to find the results of observed performance metrics, every set of experiments is executed for ten times.

*MS*. MS speed is fixed at 40 km/h.

*Query and Response*. Size of query and response data packet is assumed to be 32 byte and 256 byte, respectively. Query arrival distribution follows an exponential distribution with a mean of 30 seconds. We have defined two different deadline values for queries: *shorter* and *longer*. *Shorter deadline value* is set as expected minimum data transfer time for the distance equals to half of monitored field height. Similarly *longer deadline value* is chosen as expected minimum data transfer time from one route end to sensors located at the furthest point of the monitored field. For the current setting of simulation experiments, these values are calculated about 17 and 74 seconds for shorter and longer deadlines, respectively. Target sensors of queries are selected randomly.

### 4.2. Performance Metrics

 Performance metrics are as follows.

- *Generated Query*: Number of queries created by remote central during the simulation run time.
- *Submitted Query*: Number of queries submitted to WSN by MS.
- *Rejected Query*: Number of queries which could not be submitted to WSN due to some reason (short deadline, nonexisting connection to target sensor, empty battery, etc.).
- *Received Query*: Number of queries whose response arrives at MS on time.
- *Missed Deadline*: Number of queries whose response could not get to MS before the deadline exceeds.
- *Successful Query Ratio*: Ratio of Received Query to Generated Query.
- *Network Life Time*: Duration between the time that simulation begins and the time that any sensor's battery power gets lower than a specified level. The levels are sliced as 10% of the starting battery capacity.
- *Average Energy Consumption Per Submitted Query*: Average amount of energy consumed for forwarding query and response messages in WSN during simulation time.

For a given query generation rate distribution, any scheduling algorithm is subject to similar number of queries. However, the number of submitted query will depend on different parameters such as the current network connections, sink position, battery power of sensors, and scheduling algorithm. Ideally all generated queries should be submitted to WSN. On the other hand, to save sensor energy, scheduling algorithms can reject submitting queries whose response would not arrive on time. If the implemented scheduling algorithm is successful in selecting these kinds of queries, WSN network life would extend. Otherwise, if it fails in prediction, then system throughput will be decreased considerably. Contrary to the prediction of late response arrival, a scheduling algorithm could submit all the generated queries hoping that the responses would arrive on time.

Thus, to compare success of our algorithm, *MS with Deadline-Aware Energy-Efficient Scheduling* (MS/DES), we implemented two other data collecting approaches using *Static Sink* (SS) and *MS with Immediate Scheduling* (MS/IS). In SS approach sink is fixed and located in the middle of the monitored field to submit queries and collect responses. The other approach, MS/IS, also uses a mobile sink but with a simplified scheduling algorithm. In MS/IS, query messages are immediately forwarded to one of the gateway sensors expecting to collect responses from some gateway sensor which has the shortest path from the target sensor. Thus, MS/IS only focuses on minimizing energy consumption of response forwarding without concerning about energy consumption of query forwarding and satisfying deadline. In the simulation experiments, all parameters are set the same for these three approaches.

### 4.3. Base Experiment Results

**Figure 6:**
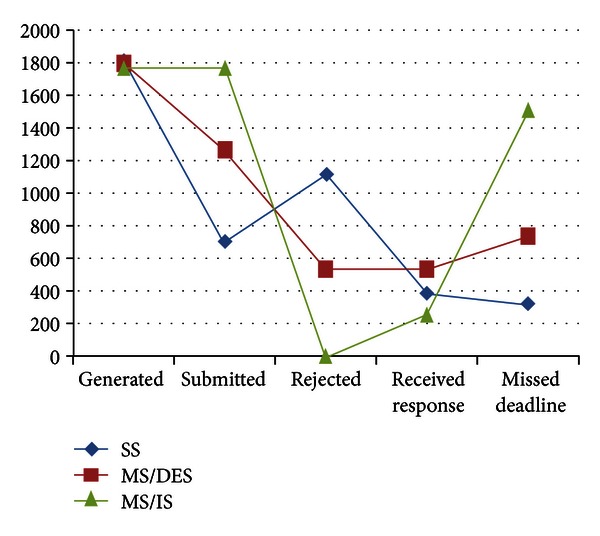
Results of various performance metrics related with query and response numbers.

**Figure 7:**
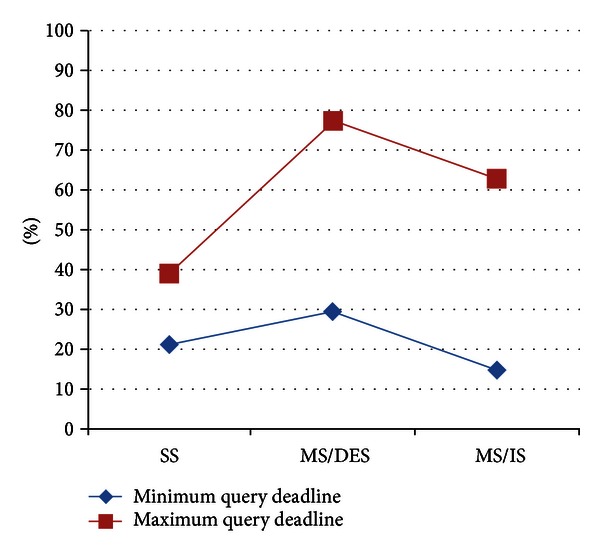
Results of Successful Query Ratio for different query deadline values.

**Figure 8:**
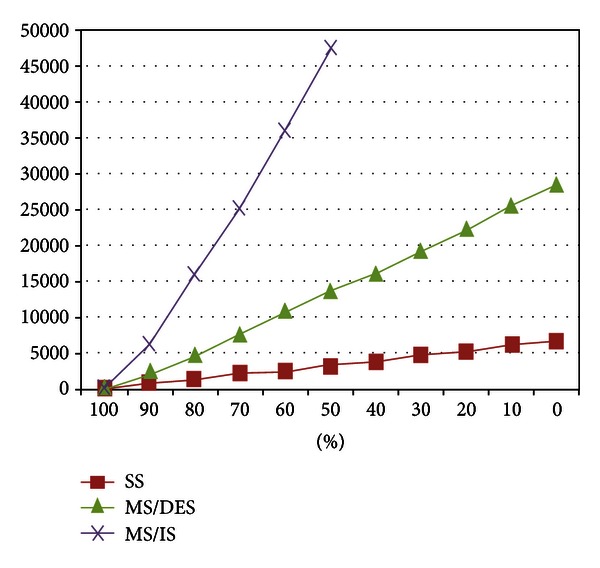
Results of Network Life Time performance metric for different battery levels.

 For the default values of parameters given in Section 4.1 with the minimum deadline value (17 sec.), we obtained the following results presented in Figures 6, 7, and 8.

In Figure 6, the effects of differences in scheduling and sink model on the results are observed. Since MS/IS does not refuse any query and submits them immediately, the numbers of missed deadlines are very high. On the other hand, MS/DES rejects submitting some queries because it calculates that they would not arrive on time; therefore, MS/DES leads to a less number of missed deadlines compared to MS/IS. SS attempts to submit every generated query, but when the sensors around the SS deplete their batteries, SS can not submit incoming queries any longer. MS/DES can submit more queries than SS and receive more responses on time than that of the others.

Figure 7 depicts the results of Successful Query Ratio metric for different query deadline values. Thanks to its prediction and rejection of queries whose responses would be possibly late, MS/DES produces the highest Successful Query Ratio for different query deadline values as well. Interestingly, SS and MS/IS have reacted to the changes in deadline contrastingly; SS functions better with less deadline values, whereas MS/IS produces better results with larger query deadlines. The reason behind these observation is that MS/IS does not have enough time to reach RCS with less query deadlines.

Figure 8 presents one perspective of the energy consumptions of the above mentioned methods: Network Life Time. In the figure, *y*-axis shows the simulation time in seconds, and *x*-axis denotes the ratio of remaining battery power to initial battery power. Time is recorded when the first sensor battery decreases to the specified battery power. SS depletes the sensors energy fast due to the nature of broadcasting via one-hop away neighbors. The sensors around the SS are the first sensors to deplete their battery since all the forwarded messages between WSN and SS pass over them. MS/IS uses sensor battery power efficiently since the responses with larger data size always follow the shortest path to the MS/IS path which minimizes the forwarding energy requirement. Contrary to MS/IS, MS/DES scarifies the battery energy usage for satisfying query deadline whenever it is required. Therefore, MS/DES consumes more energy to provide a better Successful Query Ratio as desired.

**Figure 9:**
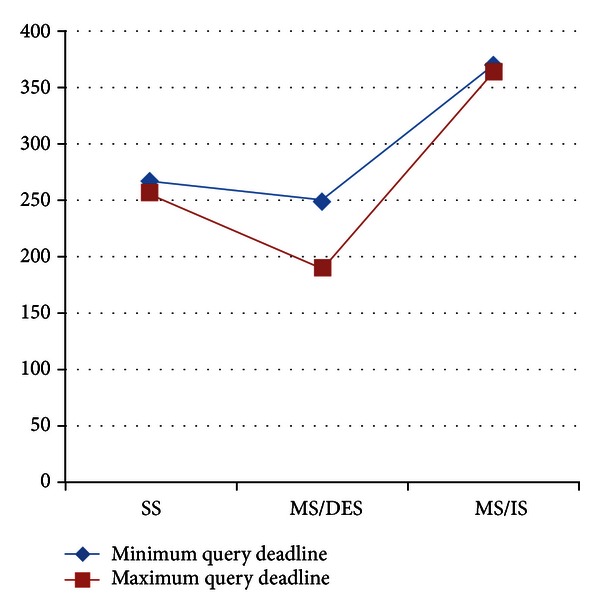
Results of Average Energy Consumption Per Submitted Query for different query deadline values.

As a second parameter to calculating energy spending of the data collection methods, we present the Average Energy Consumption Per Submitted Query in Figure 9. As depicted in the figure, SS and MS/IS almost spend the same amount of energy per query even when the deadline differs. However, MS/DES can decrease the energy consumption when query deadline is longer by adjusting the query and response path to minimize the number of hops. Thus, MS/DES is adaptive to conditions to balance the Successful Query Ratio and energy consumption.

### 4.4. Experiment Results for a Larger Deadline Value

**Figure 10:**
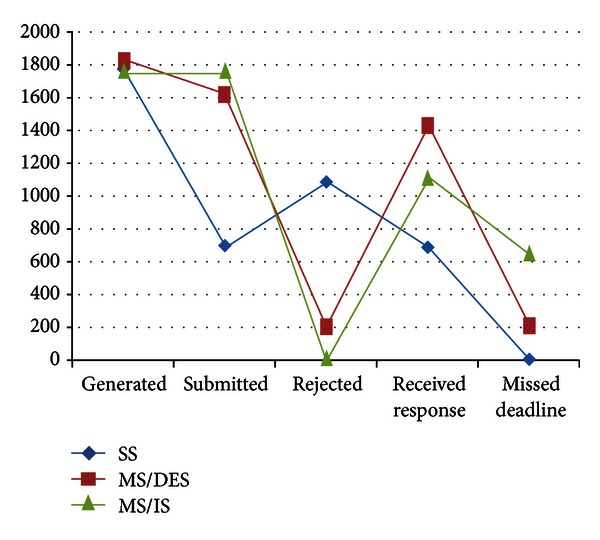
Results of various performance metrics related with query and response numbers for a larger deadline.

 For a larger value of deadline (74 sec.), we obtained the following results presented in Figure 10. The effect of having more time to collect response messages can be viewed in the increase of number of received responses and in the decrease of missed deadlines for all three data collection methods. MS/DES provides the best result for received response parameter. Furthermore, we can notice that MS/DES now rejects less number of queries which proves that MS/DES properly decides possible response arrive time. For the Successful Query Ratio given in Figure 7, MS/DES performs twice better than that of SS and 1.2 times better than that of MS/IS.

### 4.5. Experiment Results for Different Query Loads

**Figure 11:**
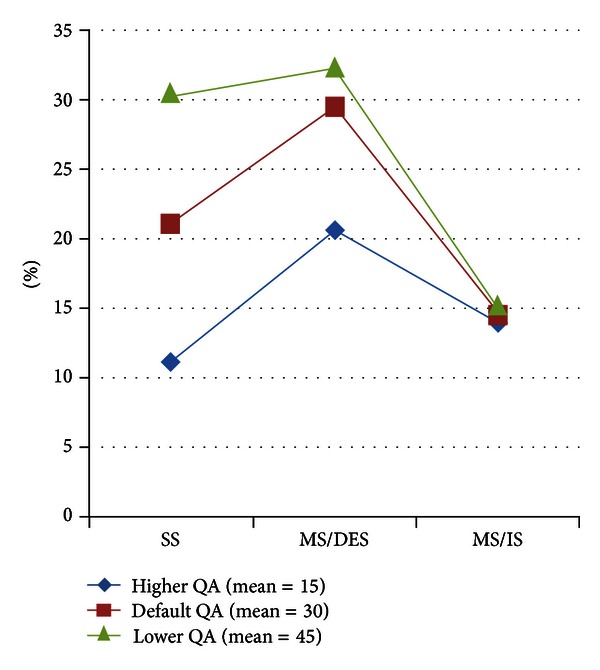
Results of Successful Query Ratio for different query loads.

**Figure 12:**
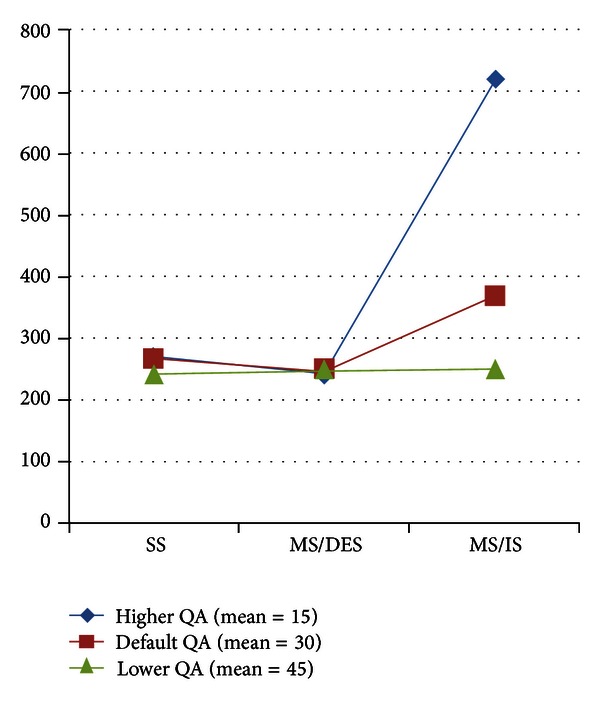
Results of Average Energy Consumption Per Submitted Query for different query loads.

 Query Arrival Rate (QA) follows exponential distribution with a mean value 30 as default. To simulate different query loads on WSN, we change the QA mean value to 15 and 45. The results of Successful Query Ration and Average Energy Consumption Per Submitted Query performance metrics for different query load values are presented in Figures 11 and 12, respectively.

Since MS/IS just submits any generated query immediately expecting to collect the response whenever MS/IS passes by the RCS, MS/IS does not react to changes in QA when the Successful Query Ration metric is observed. However, SS produces poor success query ratio when QA is high, because, for a fixed simulation time, with a higher query arrival rate, SS needs to submit more queries which leads to early battery depletion of surrounding sensors. As a result, all forthcoming queries are to be rejected. With lower QA, SS can submit more of them via its surrounding sensors before their batteries get empty. MS/DES presents better results for Successful Query Ration metric with lower QA. However, MS/DES outperforms the other approaches for all different query arrival rates.

For Average Energy Consumption Per Submitted Query performance metric, MS/IS consumes different amount of energy, whereas MS/DES and SS consume similar amount of energy when a different level of QA is applied. Since MS/IS immediately submits incoming queries to the nearest sensor and expects responses to arrive to the route via the shortest path, the difference in Energy Consumption Per Submitted Query occurs only in the query path. When there are more queries in a higher QA, the differences are increased.

On the other hand, since SS is located at the center of WSN and query and response paths are the same mostly, Average Energy Consumption Per Submitted Query is not affected by the different query load. For MS, scheduling of queries is based on the algorithm which aims to minimize the energy consumption for each query, MS/DES can sustain the same level of energy spending successfully. Thus, even MS/DES is not fixed at a location, it could accomplish similar level of success in Average Energy Consumption Per Submitted Query performance metric as SS.

## 5. Conclusions

 This paper introduced a scheduling algorithm for the location-based queries in WSN with a mobile sink following a deterministic mobility pattern. In WSN, Multihop communication pattern is used to disseminate the queries and the responses. The queries have associated with deadlines. The proposed scheduling algorithm aims at maximizing the number of successful queries and reducing the sensors' energy expenditure due to Multihop communication by exploiting deterministic sink mobility. For this reason, before submitting queries, the scheduling algorithm selects the release and collect sensors such that two important performance requirements can be met: the energy required to forward data packages should be minimum, and the response arrival time should not exceed the specified deadline.

We also simulated two data collection methods for the sake of comparison, namely, SS and MS/IS. We conducted extensive simulation tests, and the obtained results show that our scheduling algorithm can attain more successful queries with less amount of energy even when query load and deadline change.

As a future work, we would like to extend and adapt the algorithm to different mobility models other than linear route. We plan to apply some heuristics such as Ant Colony Optimization techniques to decide minimum energy consuming paths while MS decides its own route.

## Figures and Tables

**Algorithm 1:**
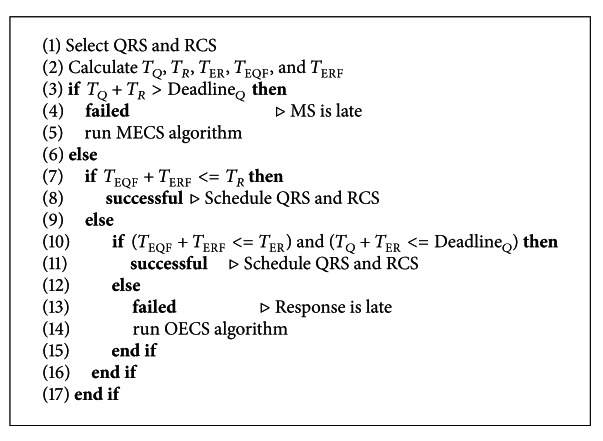
Calculate Least Energy Consuming Schedule.

**Algorithm 2:**
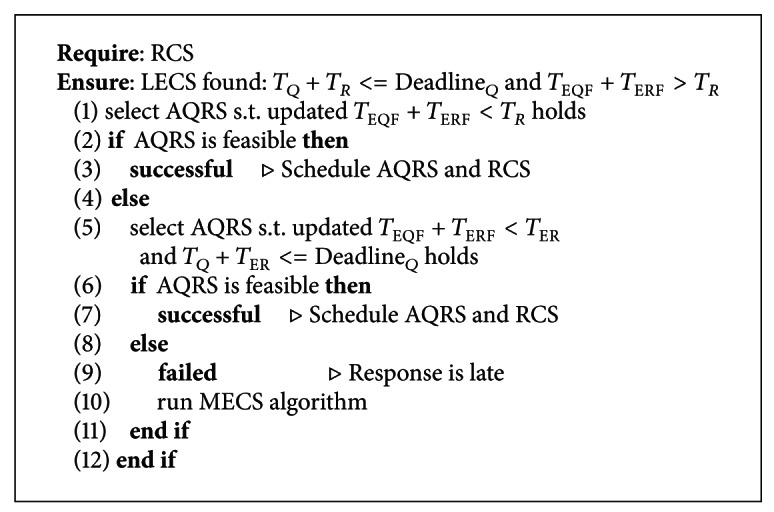
Calculate Optimum Energy Consuming Schedule.

**Algorithm 3:**
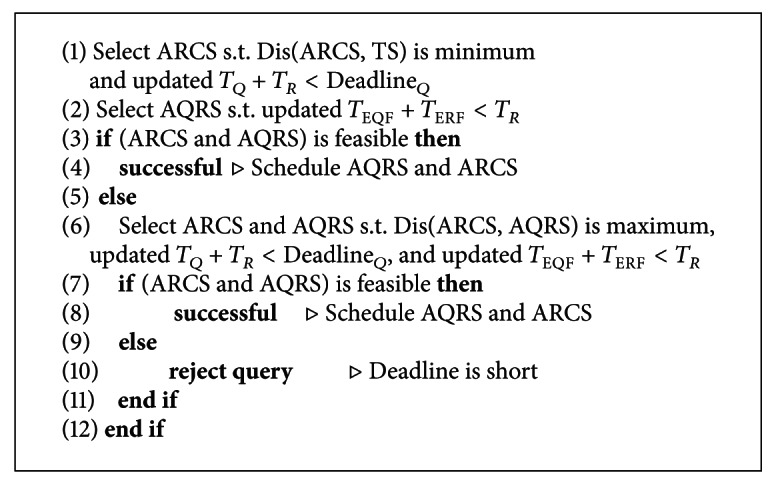
Calculate Maximum Energy Consuming Schedule.

**Table 1:** Simulation parameters and default values.

Parameters	Definition	Default setting
*W*	Width of monitored field	1000 m
*H*	Height of monitored field	500 m
*N*	Number of sensors	1771
T	Sensor topology	Grid
ST	Simulation time	54000 s (15 h)
RT	MS route location	Horizontal center
RR	Radio range	50 m
DR	Data transfer rate	256 Kb/s
EC	Data transfer cost	50 nJ/bit
BP	Initial battery power	0.01 J
QP	Query processing time	0.1 s
PC	Query processing cost	100 nJ
MS	MS speed	40 km/h
QS	Query size	32 Byte
RS	Response size	256 Byte
QA	Query arrival rate	Exp. (mean = 30 s)
DL	Deadline	min. 17 s/max. 74 s
